# Effectiveness of Novel Sympathetic Nerve Entrapment Point Injections for Chronic Migraine: A Pilot Study

**DOI:** 10.3390/life14010057

**Published:** 2023-12-28

**Authors:** Jeong Won Seong, Yuntae Kim, Dong Rak Kwon, Cheol-Jung Yang, Levent Özçakar

**Affiliations:** 1 Department of Family Medicine, Sarang Tong-sa Research Center, 2nd Floor, 477, Jinnyangho-ro, Jinju-si 52686, Republic of Korea; tongsaresearch@gmail.com; 2 Department of Physical Medicine and Rehabilitation, Soonchunhyang University Cheonan Hospital, Soonchunhyang University College of Medicine, 31 Suncheonhyang 6-gil, Dongnam-gu, Cheonan-si 31151, Republic of Korea; simon108@naver.com; 3 Department of Rehabilitation Medicine, Muscle Research Center, Catholic University of Daegu School of Medicine, 33 Duryugongwon-ro 17-gil, Nam-Gu, Daegu 42472, Republic of Korea; 4 Department of Orthopedic Surgery, Bonetouch Orthopaedic Clinic, 262, Godeok-ro, Gangdong-gu, Seoul 05269, Republic of Korea; cjyangosdr@gmail.com; 5 Department of Physical and Rehabilitation Medicine, Hacettepe University Medical School, Hacettepe, Tıp Fakültesi, Altındağ/Ankara 06230, Turkey; lozcakar@yahoo.com

**Keywords:** headache, migraine, splenius capitis, saline, injection

## Abstract

No studies to date have investigated the ability of sympathetic nerve entrapment point saline (SNEP) injections to achieve long-term pain relief in patients with migraine. Therefore, this study aimed to investigate the safety and long-term efficacy of repeat splenius capitis (SC) SNEP injections in patients with migraine (with/without tension-type headache). This retrospective, single-arm study included 12 patients with migraine. Isotonic saline was injected into their SC approximately six times for 3 months. Headache frequency, duration (hour/week), intensity (using the visual analog scale), and quality of life (using the Headache Impact Test-6) were assessed during the follow-up visits for up to 24 months after the first injection. Changes before and after treatment were assessed using repeated-measures analysis of variance. Significant reductions in headache frequency, duration, and intensity were observed at all assessment points after SNEP injections when compared with the baseline values (*p* < 0.05), while the patients’ headache-related quality of life also improved. Treatment was continued for up to 3 months to maintain these improvements, and no worsening of status or adverse effects were observed in any of the patients over the following 24 months. Our results show that SNEP injections may offer persistent, substantial, and clinically relevant benefits in patients with migraine.

## 1. Introduction

Evidence-based data on clinical signs and diagnostic test results indicate the involvement of the trigeminovascular system (including the autonomic system dysfunction) in patients with migraine [1,2,3,4,5,6,7,8]. This is also supported by observations of autonomic symptoms that can manifest during migraine attacks, such as nausea, vomiting, diarrhea, cutaneous vasoconstriction, vasodilation, piloerection, diaphoresis, photophobia, and abnormal pupillary reaction [9]. However, the precise pathophysiological mechanisms underlying involvement of the sympathetic nervous system in migraine remain unclear [10]. Afferent projections from the trigeminal ganglion converge with inputs from the C1-3 nerve roots and their branches, the O-C3 joints, the alar and transverse ligaments, the pre- and postvertebral muscles (e.g., trapezius, sternocleidomastoid), the cervical dura mater, and vertebral/carotid arteries before synapsing on second-order neurons on the trigeminal cervical complex, which encompasses the trigeminal nucleus caudalis and the dorsal horn of the upper cervical spinal cord (C1–C2) [9].

Nevertheless, cervical sympathetic nerves have been shown to play an important role in the process of neurogenic dural vasodilation in rats [11]. In addition, a recent functional neuroimaging study identified potential important alterations in the hypothalamic connectivity across the migraine phases [12], in agreement with a pivotal role of the hypothalamus in the regulation of migraine-related head pain [13]. To this end, inhibiting the vasodilatory effects of sympathetic neurotransmitters may represent an effective therapeutic approach to migraine. Moreover, although calcitonin-gene-related peptide monoclonal antibodies [14] and botulinum toxin injections [15] are used for migraine treatment, they are not always effective and are usually applied after several conservative attempts fail to improve patients’ complaints. Furthermore, adverse event profiles, cost, and patient acceptance issues are also problematic [16]. As such, there is definitely a need for novel (and preferably simple) migraine therapies.

According to the “Tong-sa hypothesis” (i.e., the “nerve entrapment point” hypothesis) [17,18,19], membrane hyperexcitability may inappropriately excite a nerve, leading to varying types of pain. Twisting or traction of the ramus communicans can also occur when sympathetic nerve entrapment syndrome (SNES) occurs due to a change in the alignment between two adjacent vertebral bodies in the thoracic and lumbar regions of the spinal cord (T1 to L2) [17,18,19]. In this context, the sympathetic nerve entrapment point (SNEP) is defined as the site of SNES. SNEP injection treatment refers to the intramuscular injection of isotonic saline into the problematic paraspinal deep muscles related to the corresponding internal organ dysfunction. This treatment is thought to ultimately resolve the focal excitability of the spinal nerves, including the sympathetic preganglionic fibers [17,18,19]. Notably, considering the local anatomy—i.e., origin, insertion, and fiber extension—the splenius capitis (SC) muscle shows the strongest physical torsion in the upper thoracic spine for possible induction of SNES at the superior cervical ganglia, which is formed by the union of four sympathetic ganglia of the cervical spinal nerves C1–C4. A previous report showed that cluster headache can be alleviated via a simple method, i.e., SNEP injection, via injecting isotonic saline into the splenius capitis muscle or the paraspinal deep muscles (T1–4 levels). The mechanism is thought to de-excite the spinal nerves, including the sympathetic preganglionic fibers. The hypothalamus may become damaged by reduced intracranial blood flow and constricting blood vessels due to abnormal excitement of the superior cervical ganglion by the mechanical hyperexcitable splenius capitis muscle or the paraspinal deep muscle. The eventual malfunction may trigger cluster headache because of the abnormal “alarm sparks” from the hypothalamus [19]. To the best of our knowledge, no study has reported the use of SNEP injections for treating patients with migraine.

In light of the above-mentioned issues, this study aimed to explore the long-term efficacy of novel SNEP injections of isotonic saline into the SC muscle in patients with migraine.

## 2. Materials and Methods

### 2.1. Participants

In this pilot study, we identified potential participants via a retrospective chart review of patients who visited the Jinju Sarang Tong-sa Research Center, Republic of Korea, between March 2016 and December 2019. Clinical and video-based information on the patients’ pain levels were evaluated. This study was approved by the Ethics Committee of Soonchunhyang University Cheonan Hospital (protocol code 2020-04-032). Given the retrospective nature of this study, the need for informed consent was waived.

We evaluated 66 patients with headache based on the specific inclusion/exclusion criteria for injections at the active SC tender points identified during physical examination. The inclusion criteria were as follows: (i) patients aged ≥ 18 years who had experienced headaches at least two to five times per month for >12 months; (ii) those who were diagnosed with chronic migraine with/without tension-type headache (TTH) according to the International Classification of Headache Disorders 3rd edition, beta version [20]; (iii) those who had tender points in the SC muscles, with target areas coinciding with the site of migraine with/without TTH pain; and (iv) those who had minimal response to pharmacotherapy (≤10% reduction from the baseline, in the number of days/month with migraine during a 3 month period) [21].

We excluded patients if they had the following conditions: (i) headache caused by organic disorders (e.g., subarachnoid or cerebral hemorrhage, cerebral embolism or thrombosis, and vascular malformation), except migraine without aura; (ii) needle phobia; (iii) any contraindication for topical treatment or injection of trigger points; (iv) history of neurological, psychiatric, and cognitive problems; (v) and being already under migraine prophylaxis at the first visit. The patients enrolled in the study were thoroughly verified to be devoid of any musculoskeletal problems, fibromyalgia, or rheumatic diseases that could potentially influence headache.

### 2.2. SNEP Injection

SNEP injections were administered for approximately three months according to the following schedule: twice weekly in the first two weeks, once weekly in the next two weeks, every other week for the next month, and monthly for the remaining period.

#### 2.2.1. SC Injection Technique

**Figure 1:**
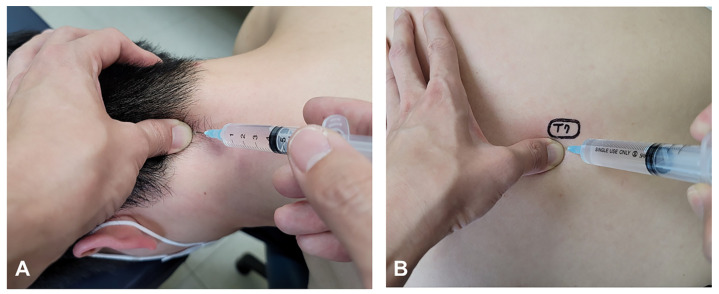
(**A**) Sympathetic nerve entrapment point (SNEP) injection of 4.0 mL of isotonic saline into the splenius capitis muscle using a 23 gauge, 1 inch needle. (ShinChang Medical Corp, Gumi, Republic of Korea) (**B**) Deeply localized (DL) SNEP injection. The tender points in the T7/T8 interspinous space are identified after the patient is completely relaxed. Isotonic saline (4 mL) is then injected at a point half a finger-breadth from the spinous process using a 23 gauge, 1 inch needle.

Patients were seated in a chair with their foreheads on the examination table to reduce posterior neck muscle tension. We identified a tender point halfway between the C2 spinous and mastoid processes. Pressure was applied to the SC muscles for 10 s to elicit reference pain. Subsequently, we selected the active pain-eliciting SC muscles, which are most commonly observed in patients with migraine. To reduce the distance between the skin and SC muscle inside the fascia, gradual and gentle pressure was applied to the skin and subcutaneous tissue using the thumb of the clinician’s left hand. Thereafter, the skin was sterilized using a topical alcohol solution. Then, the clinician held a 23 gauge, 1.0 inch needle syringe containing sterile isotonic saline (NaCl, 0.9%) in his right hand and inserted the needle at the nearest distance possible to his left hand in order to facilitate the advancement of the syringe into the muscle at an approximate angle of 30°. Immediately after the skin was pierced, the syringe was moved forward, with the clinician holding the syringe in a relaxed manner to feel the outer surface of the fascia since it has a denser structure than the subcutaneous tissue. Therefore, the outer surface of the fascia could be felt when the tip of the needle touched it. Upon perceiving the denser structure, a slight force was gently applied so as to penetrate approximately 2 mm into the fascia, after which 4 mL of isotonic saline was injected into the muscle. During injection, the clinician felt the spread of isotonic saline and the SC muscle texture with his left hand (Figure 1A).

#### 2.2.2. Deep Injection Technique

Deeply localized (DL) SNEP injection refers to intramuscular injection into the transversospinales of the selected segment (corresponding to the sympathetic symptoms) in a patient who is completely relaxed and positioned with their face down. We identified the needle insertion site by determining the precise location and tender point in the spine; thereafter, the procedure can be easily performed (Figure 1B).

### 2.3. Research Outcomes

The primary outcome was the number of migraine attacks when compared with that at baseline and for the preceding weeks. The secondary outcomes included headache duration (hours/week), headache intensity, and headache-related quality of life as assessed using the Headache Impact Test-6 (HIT-6) [22]. Headache intensity was assessed at each visit using a 10 cm horizontal visual analog scale (VAS) ranging from 0 (“no pain”) to 10 (“the worst imaginable pain”). The VAS assessment in this study considered average patient pain over the 24 h before assessment [23]. The minimal clinically important difference for the VAS is estimated to be 3 cm [24].

We requested the patients indicate the highest intensity of headache experienced during the previous and current weeks (VAS1 and VAS2, respectively). The Korean version of the HIT-6 covers multiple aspects, including pain, social functioning, role functioning, vitality, cognitive functioning, and psychological distress, and it was developed to measure a wider spectrum of headache-induced disability [25]. We scored each item on a five-point Likert scale (6 = never, 8 = rarely, 10 = sometimes, 11 = very often, and 13 = always). The total score ranged between 36 and 78, wherein higher scores indicated worse headaches. For interpretation, we categorized the HIT-6 scores into four groups as follows: ≤49, little or no impact of headache; 50–55, some impact; 56–59, substantial impact; and ≥60, severe impact. The Cronbach’s α coefficient for the HIT-6 was 0.85. Moreover, we assessed the dose per week in patients receiving medications for migraine and recorded the number of pre- and post-treatment acetaminophen, non-steroidal anti-inflammatory, or triptan drug pills consumed. Outcome measurements were performed before injection (baseline, assessment point 1) and 1 month (assessment point 2), 2 months (assessment point 3), 3 months (assessment point 4), 12 months (assessment point 5), and 24 months (assessment point 6) after the first injection by a blinded investigator.

### 2.4. Statistical Analyses

Continuous variables are represented as median and interquartile range. To assess changes (before vs. after treatment), we performed a repeated-measures analysis of variance and conducted a trend analysis for the repeated-measures variables. To compare data that were not normally distributed, we used non-parametric Friedman’s repeated-measures analysis and corrected *p*-values for each correspondence. A *p*-value of <0.50 was considered statistically significant. SPSS version 25.0 (IBM SPSS, Armonk, NY, USA) was used for all statistical analyses.

## 3. Results

**Figure 2:**
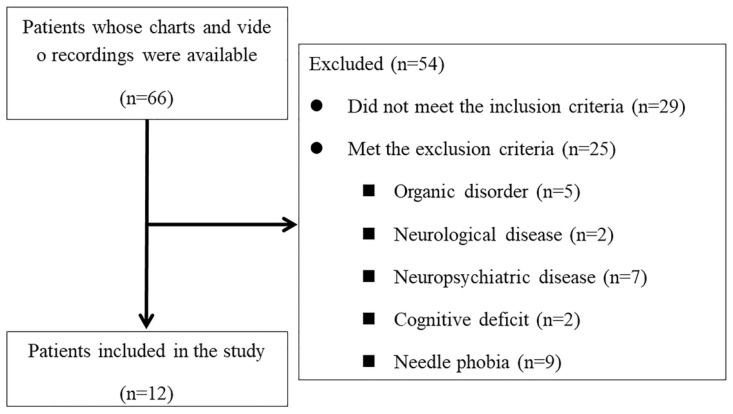
Flowchart of the patient selection process.

Of the 66 patients, we eventually included 12 (3 men, 9 women; median age, 42.5 years; median symptom duration, 73 months) in the final analysis (Figure 2, Table 1). We excluded 54 patients who were not followed until 24 months after the first injection. The median duration of treatment was 40 days, and the average number of treatment sessions was six. Overall, nine patients were diagnosed with migraine, while three were diagnosed with mixed migraine and TTH. For patients with migraine and dizziness, T1–T4 level DL SNEP injection was the main treatment modality. For patients with migraine associated with dyspepsia or worsened symptoms during menstrual periods, T6–T7 level DL SNEP injection and T9–T12 level DL SNEP injection were the treatment modalities, respectively. Overall, nine patients received treatment only to the SC muscle, whereas the others received additional DL SNEP injection treatment. We did not observe any side effect during the injections. In addition, no adverse effect was observed in any of the patients during the treatment period and follow-up.

**Figure 3:**
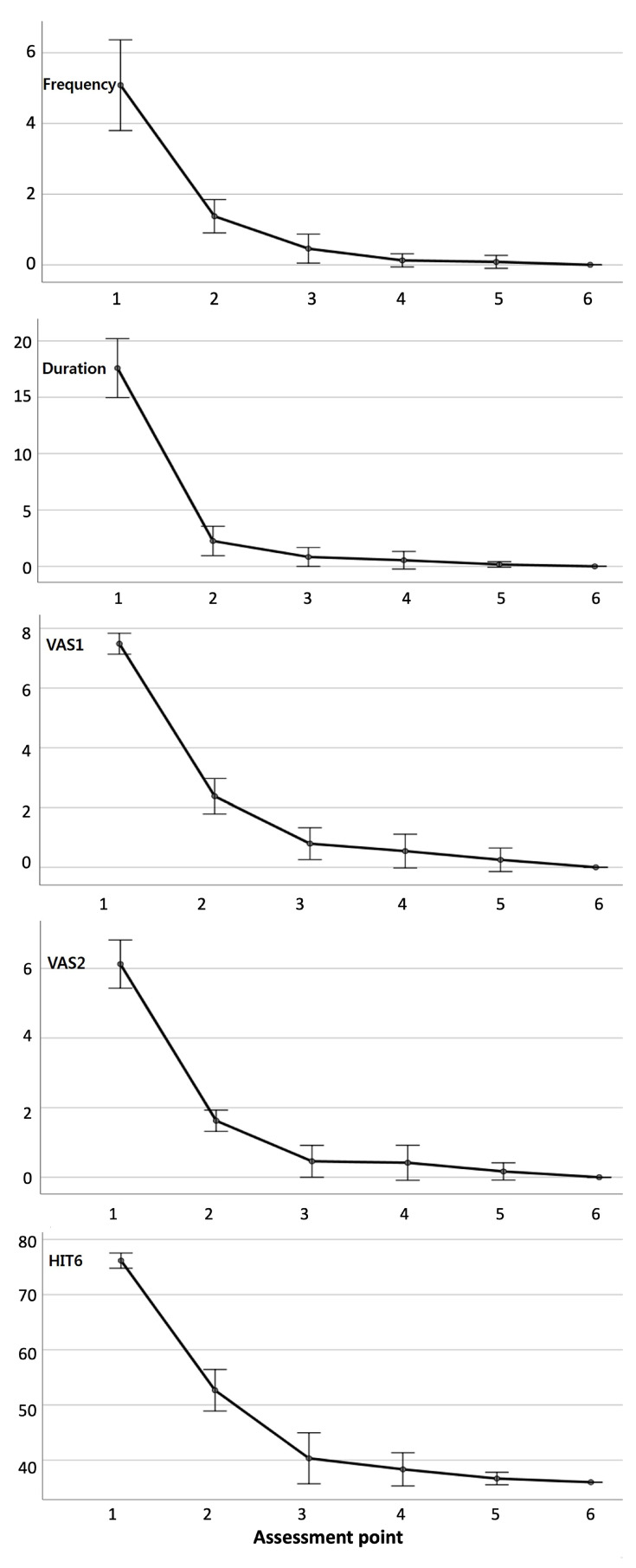
Changes according to assessment points during the 24 month follow-up of 12 patients. Compared with the baseline values, statistically significant reductions in the headache frequency and duration, highest intensity of headache experienced during the previous and current weeks (VAS1 and VAS2, respectively), and Headache Impact Test-6 scores were observed at all assessment points following SNEP injection (*p* < 0.001). Assessment point 1, at baseline (before injection); assessment point 2, at 1 month; assessment point 3, at 3 months; assessment point 4, at 6 months; assessment point 5, at 12 months; assessment 6, at the 24 months follow-up visit after the first injection. Abbreviations: VAS, visual analogue scale; SNEP, sympathetic nerve entrapment point.

Compared with the baseline values, significant reductions in headache frequency and duration, VAS1, VAS2, and HIT-6 scores were observed at all assessment points following SNEP injection (*p* < 0.001). Additionally, all patients exhibited improvement in pain that exceeded the minimal clinically important difference on the VAS post-intervention. We also observed significant improvements during the remaining part of the study period, when compared with the first-month results (*p* < 0.05). Treatment was continued for up to three months to maintain these improvements. Following the 3 month treatment period, no worsening of symptoms was observed in any patient over the 24 months post-intervention. There was no statistically significant difference between patient parameters in the last month of treatment and those at 24 months post-intervention (Table 2, Figure 3). Table 3 summarizes the parameters at each evaluation point.

Most patients (n = 10) were under long-term acetaminophen, non-steroidal anti-inflammatory, or triptan treatment. Moreover, they reported that the administration of 2–3 pills/day failed to improve their headache during the attacks. However, during the post-injection telephone interviews, most patients (n = 10) did not report headaches even without any medication.

## 4. Discussion

This retrospective study examined the safety and long-term efficacy of novel SC SNEP injection using isotonic saline in patients with migraine. Our results indicate that all patients had favorable outcomes following an average of six treatment sessions and during the 24 month follow-up period. Repeated SC SNEP injection treatments using isotonic saline were shown to have persistent and clinically relevant benefits for migraine prophylaxis, encompassing reductions in headache frequency, duration, and intensity as well as improvements in headache-related quality of life. These results highlight the ease, convenience, and safety with which the SC SNEP procedure can be performed in an outpatient clinic. Therefore, clinicians can perform the procedure without concerns of major adverse effects, which are common when administering interventional drugs in daily outpatient procedures. In addition, these results demonstrate the need to obtain data related to the long-term effects of repeat SC SNEP injection in patients with migraine.

SC SNEP injection involves an intramuscular injection into the SC muscle of a completely relaxed patient who has been positioned with their forehead on the examination table. Clinical studies have suggested that the effects of intramuscular injections are generally related to the mechanical rather than the pharmacological effects of the intervention [26]. Further, for the treatment of migraine, the most common muscles selected for trigger point injections include the trapezius, sternocleidomastoid, and temporalis. Other muscles that may also feature trigger-point-induced referred pain to the head and neck include the cervical paraspinal muscles, masseter, levator scapulae, frontalis, and occipitalis. In this study, we identified the SC as the most important key muscle and we considered that its anatomy plays a very important role in the balance of the autonomic nerves, unlike the other muscles around the neck [15].

Injection of corticosteroids with/without a local anesthetic is reportedly not superior to that of isotonic saline for myofascial pain syndrome [27,28]. Moreover, repeat injections of local anesthetic can cause muscle toxicity. The effectiveness of botulinum toxin wanes after three months, making repeat injections essential. This results in a significant economic burden despite the fact that botulinum toxin is a useful clinical alternative to conventional treatment of intractable myofascial pain associated with temporomadibular disorder [29].

Therefore, we selected isotonic saline for this study, which is affordable and has not been reported to have any associated side effects even after repeat injections. The mechanism of the favorable effect of the treatment may be attributed to increased oxygen and blood supply to the local area due to reactive hyperemia. Reactive hyperemia is currently considered the mechanism underlying the effects of massage and ischemic compression [30]. Moreover, a pain-free period may disrupt the repetitive output from the neural circuits established among the nociceptors, central nervous system, and motor units [31,32].

Our findings also demonstrated the long-term efficacy of SC SNEP injection for migraine prophylaxis. However, they do not completely correlate with those of a previous study [33], and this inconsistency may be attributed to differences in the treated SC muscles and treatment concepts. The SC is a pericranial muscle and originates from the spinous process of the C7–T4 vertebrae. The insertion points are directed upward and laterally, extending to the occipital bone immediately below the lateral one-third of the superior nuchal line, as well as the mastoid process of the temporal bone and underneath the sternocleidomastoid muscle. Accordingly, the SC displays the strongest physical torsion for the induction of tender points and SNES at the superior cervical ganglia.

We based the SNEP injection treatment in this study on the hypothesis that the ramus communicans can be twisted or pulled by a change in the alignment between the two adjacent vertebral bodies [14]. The presence of focal ischemia followed by the compression of the spinal nerve trunk in the intervertebral foramen and vasa nervorum of the sympathetic preganglionic fibers in the spinal nerve trunk may decrease the production of ATP. This may, in turn, result in a failure of the Na+/K+ ATP pump, leading to an elevation of the extra-membranous K+ concentration, which in turn may increase the resting membrane potential, thereby reducing the threshold potential of nerve cells [34,35] and leading to membrane hyperexcitability. Hyperexcited vasomotor sympathetic preganglionic fibers become sensitive even to small stimuli, eventually producing abnormal excitation signals. The increased production of norepinephrine at sympathetic postganglionic fiber terminals activates alpha-1 receptors on vascular smooth muscles, thus causing vasoconstriction, which eventually reduces the blood flow to internal organs (such as the stomach) and tissues [36].

Previous reports demonstrated that upper gastrointestinal symptoms were improved by performing SNEP injection in the T5-T7 multifidus area. Upper gastrointestinal symptoms seemingly occurred because the entrapped sympathetic nerve fibers were overstimulated at the corresponding level. Therefore, by performing SNEP injection on the multifidus muscle, intervertebral pressure and constriction of the vasa nervorum were alleviated, which improved the compression of the sympathetic preganglionic fibers and increased the blood flow to the sympathetic nerves. As such, the overexcited sympathetic nerves were normalized [19,37]. Sympathetic nerves that innervate the stomach arise at the T6–9 level, and postganglionic sympathetic neurons use norepinephrine as the main neurotransmitter [38]. When a sympathetic nerve becomes excited, it inhibits gastrointestinal motility and induces vasoconstriction by acting on vascular smooth muscle to regulate gastric blood flow [38,39]. Sympathetic preganglionic fibers are located in the intervertebral foramen of the corresponding level. When this point is overexcited by various stimuli, signals are excessively transmitted to the postganglionic fibers, which lead to excess secretion of norepinepherine from the terminal part of the nerve. This causes a decrease in the motility of the intestine and a temporary decrease in the blood flow due to vasoconstriction [17,38]. Consequently, this excess excitation of the sympathetic nerves leads to the failure of proper regulation, resulting in an imbalance in the autonomic nervous system, which might cause nausea and vomiting [40,41]. SNEP injection treatment principally targets internal organ dysfunction and ultimately aims to resolve the focal excitability of spinal nerves, including sympathetic preganglionic fibers [36]. Neuroanatomically, the sympathetic preganglionic fibers carry autonomic efferent signals from the C8–T4 intermediolateral cell columns that travel via the ipsilateral ventral roots of the C8–T4 spinal segments. They eventually reach the superior cervical ganglia, where postganglionic fibers receive the signals to innervate the vascular smooth muscles of the head and neck. In the intervertebral foramen, selectively hyperexcited sympathetic preganglionic fibers at C8–T4 may actively send excitability signals to sympathetic postganglionic fibers by increasing the secretion of norepinephrine, which binds to alpha-1 receptors in the vascular smooth muscles of the head and neck, thus resulting in vasoconstriction. Herein, SNEP injection into the SC reduces the intervertebral pressure and dilates the vasa nervorum, thus facilitating blood supply to the nerves through direct innervation of the autonomic nervous system [37]. Consequently, the blood supply to the sympathetic preganglionic fibers is enhanced, and the level of norepinephrine at the nerve terminals is normalized. Similarly, blood flow to the head and neck may increase and eventually stabilize the parasympathetic fibers.

With a follow-up of 24 months, this novel study is one of the longest trials to use a rigorous method to determine the efficacy of true SC SNEP injections. We obtained data regarding the mean headache frequency, duration, and intensity as well as HIT-6 scores for each of the six treatment sessions to evaluate the therapeutic and curative effects of SC SNEP injection lasting up to 24 months. However, a long observation period has its limitations. For example, the rate of patient dropout is likely to be high, and patients may fail to report other treatments during the follow-up period. To minimize potential bias, we reduced dropouts by establishing a good rapport with the participants and maintaining close contact through telephone calls, text messages, and e-mails during the follow-up.

This study had some limitations, including its retrospective design and small sample size. While the retrospective design precluded any prospective capture of adverse effects associated with SC SNEP injection, the small sample size also precluded comparison among different headache types. Moreover, our study lacked a sham or another control group to directly compare SC SNEP injection with other standard migraine interventions or pharmacotherapies. In this sense, the risk of interpretation bias could be high. Additionally, documented patient- or physician-reported outcomes may be subject to bias as well. The subjective measure of efficacy was another limiting factor, although this is similar to those reported in other studies that have used pain scores to assess the efficacy of SC SNEP injection. Needless to say, the use of imaging guidance during the interventions would have also facilitated the study protocol. Nonetheless, the long-term follow up (over a 24 month period) could, on the other hand, be considered as the most important strength of this small study.

## 5. Conclusions

This pilot study demonstrated that repeat injections of isotonic saline into the SC muscle may be an effective and safe method to relieve headache intensity and improve quality of life among patients with chronic migraine. Specifically, the therapeutic effects persisted over a long period (up to 24 months) without recurrence. However, comparative studies with larger samples are required to confirm these preliminary results and to further design possible treatment algorithms for different types of headaches.

## Figures and Tables

**Table 1:** Patient characteristics.

Total	n = 12	
Men	n = 3	
Women	n = 9	
Age (years)	median = 42.5	(IQR, 30.8–55)
Men	median = 33	(IQR, 30–62)
Women	median = 45	(IQR, 33–55)
Types of headache		
V	n = 9	
V + M	n = 3	
Duration of headache (month)	median = 73	(IQR, 17.3–121.7)
Duration of treatment (day)	median = 40	(IQR, 30–71.5)
Number of treatments	median = 6	(IQR, 5.3–7.8)

Abbreviations: IQR, interquartile range; V, visceral headache; M, musculoskeletal headache; IQR, interquartile range.

**Table 2:** Comparison of the assessment parameters during the 24 month follow-up period.

	Time	Mean ± SD	95% CI	*p*-Value	*p*-Value				
**Frequency** **(days/week)**	**Baseline**	5.08 ± 2.02	3.80–6.37	<0.001	ref.				
	**1 month**	1.38 ± 0.74	0.90–1.85		**<0.001**	ref.			
	**3 months**	0.46 ± 0.65	0.05–0.87		**<0.001**	**<0.001**	ref.		
	**6 months**	0.13 ± 0.29	(−0.06)–0.31		**<0.001**	**<0.001**	0.275	ref.	
	**12 months**	0.08 ± 0.29	(−0.10)–0.27		**<0.001**	**<0.001**	0.279	1.000	ref.
	**24 months**	0.00 ± 0.00	0.00–0.00		**<0.001**	**0.001**	0.480	1.000	1.000
**Duration** **(hours/week)**	**Baseline**	17.58 ± 4.12	14.96–20.20	<0.001	ref.				
	**1 month**	2.25 ± 2.05	0.95–3.55		**<0.001**	ref.			
	**3 months**	0.83 ± 1.32	(−0.01)–1.67		**<0.001**	**0.011**	ref.		
	**6 months**	0.54 ± 1.23	(−0.24)–1.33		**<0.001**	**0.004**	0.406	ref.	
	**12 months**	0.17 ± 0.39	(−0.08)–0.41		**<0.001**	**0.023**	0.526	1.000	ref.
	**24 months**	0.00 ± 0.00	0.00–0.00		**<0.001**	**0.044**	0.769	1.000	1.000
**VAS1**	**Baseline**	7.48 ± 0.55	7.13–7.83	<0.001	ref.				
	**1 month**	2.38 ± 0.93	1.78–2.97		**<0.001**	ref.			
	**3 months**	0.79 ± 0.84	0.26–1.32		**<0.001**	**0.008**	ref.		
	**6 months**	0.54 ± 0.89	(−0.02)–1.11		**<0.001**	**0.002**	1.000	ref.	
	**12 months**	0.25 ± 0.62	(−0.14)–0.64		**<0.001**	**<0.001**	0.122	1.000	ref.
	**24 months**	0.00 ± 0.00	0.00–0.00		**<0.001**	**<0.001**	0.112	0.884	0.179
**VAS2**	**Baseline**	6.13 ± 1.09	5.43–6.82	<0.001	ref.				
	**1 month**	1.63 ± 0.48	1.32–1.93		**<0.001**	ref.			
	**3 months**	0.46 ± 0.72	0.00–0.92		**<0.001**	**0.002**	ref.		
	**6 months**	0.42 ± 0.79	(−0.09)–0.92		**<0.001**	**0.003**	1.000	ref.	
	**12 months**	0.17 ± 0.39	(−0.08)–0.41		**<0.001**	**<0.001**	0.695	1.000	ref.
	**24 months**	0.00 ± 0.00	0.00–0.00		**<0.001**	**<0.001**	0.751	1.000	1.000
**HIT-6** **(range: 36–78)**	**Baseline**	76.17 ± 2.17	74.79–77.54	<0.001	ref.				
	**1 month**	52.67 ± 5.93	48.90–56.43		**<0.001**	ref.			
	**3 months**	40.33 ± 7.28	35.71–44.96		**<0.001**	**<0.001**	ref.		
	**6 months**	38.33 ± 4.74	35.32–41.34		**<0.001**	**<0.001**	0.79	ref.	
	**12 months**	36.67 ± 1.78	35.54–37.79		**<0.001**	**<0.001**	0.751	1.000	ref.
	**24 months**	36.00 ± 0.00	36.00–36.00		**<0.001**	**<0.001**	0.954	1.000	1.000

Abbreviations: SD, standard deviation; CI, confidence interval.

**Table 3:** Individual patient parameters at each assessment time-point.

	Baseline					1 Month					3 Months					6 Months					12 Months					24 Months				
	F	D	V1	V2	H	F	D	V1	V2	H	F	D	V1	V2	H	F	D	V1	V2	H	F	D	V1	V2	H	F	D	V1	V2	H
**A**	6	16	7	6	76	2	3	2	2	52	0.25	0	1	0	36	0	0	0	0	36	0	0	0	0	36	0	0	0	0	36
**B**	7	16	7	7	78	2	3	3	2	60	1	1	1	1	44	0	0	0	0	36	0	0	0	0	36	0	0	0	0	36
**C**	4	16	7	6	74	1.5	1	1.5	1	50	0.25	0.5	1	0	36	0	0	0	0	36	0	0	0	0	36	0	0	0	0	36
**D**	3	14	8	7	74	1.5	5	3	2	60	1	3	2.5	1.5	50	0.25	2	2.5	2	46	0	1	1	1	38	0	0	0	0	36
**E**	3	15	8	8	76	1	1	3	2	50	0	0	0	0	36	0	0	0	0	36	0	0	0	0	36	0	0	0	0	36
**F**	7	24	8.5	4	78	1	2	4	2	50	0	0	0	0	36	0	0	0	0	36	0	0	0	0	36	0	0	0	0	36
**G**	6	24	7.8	6	78	1.5	2	2	1.5	48	1	1	1	1	44	0.25	0.5	1	1	40	0	0	0	0	36	0	0	0	0	36
**H**	6	16	7.5	5	72	1	1	2	1	54	0	0.5	1	0	36	0	0	1	0	36	0	0	0	0	36	0	0	0	0	36
**I**	4	16	7	6	78	1	2	3	2	56	0	0	0	0	36	0	0	0	0	36	0	0	0	0	36	0	0	0	0	36
**J**	7	24	8	7	78	3	7	3	2	62	2	4	2	2	58	1	4	2	2	50	1	1	2	1	42	0	0	0	0	36
**K**	1	12	7	5	74	0	0	1	1	48	0	0	0	0	36	0	0	0	0	36	0	0	0	0	36	0	0	0	0	36
**L**	7	18	7	6.5	78	1	0	1	1	42	0	0	0	0	36	0	0	0	0	36	0	0	0	0	36	0	0	0	0	36

Abbreviations: F, frequency; D, duration; V, visual analog scale; H, Headache Impact Test-6.

## Data Availability

The data presented in this study are available on request from the corresponding author.
